# Standardised patient study to assess tuberculosis case detection within the private pharmacy sector in Vietnam

**DOI:** 10.1136/bmjgh-2021-006475

**Published:** 2021-10-05

**Authors:** Shukry Zawahir, Hien Le, Thu Anh Nguyen, Justin Beardsley, Anh Dang Duc, Sarah Bernays, Kerri Viney, Thai Cao Hung, Shannon McKinn, Hoang Huy Tran, Son Nguyen Tu, Kavindhran Velen, Tan Luong Minh, Hung Tran Thi Mai, Nhung Nguyen Viet, Ha Nguyen Viet, Van Nguyen Thi Cam, Thanh Nguyen Trung, Stephen Jan, Ben J Marais, Joel Negin, Guy B Marks, Gregory Fox

**Affiliations:** 1 Faculty of Medicine and Health, The University of Sydney, Sydney, New South Wales, Australia; 2 Woolcock Institute of Medical Research, Kim Ma, Hanoi, Vietnam; 3 The Marie Bashir Institute, Westmead Institute for Medical Research, The University of Sydney, Sydney, New South Wales, Australia; 4 National Institute of Hygiene and Epidemiology, Hanoi, Vietnam; 5 Public Health and Policy, London School of Hygiene and Tropical Medicine, London, UK; 6 Centre of Global Health, Department of Public Health Sciences, Karolinska Institute, Stockholm, Sweden; 7 Research School of Population Health, Australian National University, Canberra, Australian Capital Territory, Australia; 8 Medical Service Administration, Government of Viet Nam Ministry of Health, Hanoi, Vietnam; 9 Clinical Pharmacy, Hanoi University of Pharmacy, Hanoi, Vietnam; 10 National TB Program, Vietnam National Lung Hospital, Hanoi, Vietnam; 11 The George Institute for Global Health, Newtown, New South Wales, Australia; 12 Marie Bashir Institute for Infectious Diseases and Biosecurity and the Children’s Hospital at Westmead, Sydney Medical School, The University of Sydney, Sydney, New South Wales, Australia; 13 Faculty of Medicne and Health, The University of Sydney School of Public Health, Sydney, New South Wales, Australia; 14 South Western Sydney Clinical School, University of New South Wales, Sydney, New South Wales, Australia; 15 Woolcock Institute of Medical Research, Glebe, New South Wales, Australia

**Keywords:** tuberculosis, other study design, health services research, public health

## Abstract

**Background:**

Of the estimated 10 million people affected by (TB) each year, one-third are never diagnosed. Delayed case detection within the private healthcare sector has been identified as a particular problem in some settings, leading to considerable morbidity, mortality and community transmission. Using unannounced standardised patient (SP) visits to the pharmacies, we aimed to evaluate the performance of private pharmacies in the detection and treatment of TB.

**Methods:**

A cross-sectional study was undertaken at randomly selected private pharmacies within 40 districts of Vietnam. Trained actors implemented two standardised clinical scenarios of presumptive TB and presumptive multidrug-resistant TB (MDR-TB). Outcomes were the proportion of SPs referred for medical assessment and the proportion inappropriately receiving broad-spectrum antibiotics. Logistic regression evaluated predictors of SPs’ referral.

**Results:**

In total, 638 SP encounters were conducted, of which only 155 (24.3%) were referred for medical assessment; 511 (80·1%) were inappropriately offered antibiotics. A higher proportion of SPs were referred without having been given antibiotics if they had presumptive MDR-TB (68/320, 21.3%) versus presumptive TB (17/318, 5.3%; adjusted OR=4.8, 95% CI 2.9 to 7.8). Pharmacies offered antibiotics without a prescription to 89.9% of SPs with presumptive TB and 70.3% with presumptive MDR-TB, with no clear follow-up plan.

**Conclusions:**

Few SPs with presumptive TB were appropriately referred for medical assessment by private pharmacies. Interventions to improve appropriate TB referral within the private pharmacy sector are urgently required to reduce the number of undiagnosed TB cases in Vietnam and similar high-prevalence settings.

**Key questions**

**What is already known?**

- Tuberculosis (TB) is a leading infectious disease killer globally; control efforts are undermined by inadequate case detection and delayed diagnoses, particularly in private settings in countries with a high incidence of TB.
- Patients with potential TB frequently approach private pharmacies to access care in these TB high-incidence settings; they are often not referred for TB screening and are supplied a short-term course of broad-spectrum antibiotics, which lead to delayed case detection and enable ongoing community transmission.
- There was no geographically representative evidence estimating the magnitude of the problem regarding presumptive TB patient referrals and antibiotic supply within the private pharmacy sector in Vietnam, which responds to two-thirds of the population’s healthcare needs.

**What are the new findings?**

- Our multicentre study, using a standardised patient method, provides estimates of pharmacy referral practices for patients with presumptive TB in the northern and southern regions of Vietnam.
- Private pharmacies frequently fail to recognise TB symptoms or to refer patients to TB services, contributing to low rates of case detection; many patients with presumptive TB were inappropriately given antibiotics, including fluoroquinolones.
- These findings add to the growing evidence in the literature on limited contribution of private pharmacies to TB case finding and the appropriate use of antibiotics in community settings.

**Key questions**

**What do the new findings imply?**

- Our results demonstrate the need to improve the skills of pharmacy staff in the early recognition and appropriate referral of patients with possible TB.
- The study highlights the importance of improving antimicrobial stewardship in the community pharmacy setting and supports the WHO’s important recommendations for public–private mix approaches to strengthen TB case finding.

## Background

Tuberculosis (TB) is a leading infectious cause of death globally, and TB case numbers are estimated to increase even further due to the disruptions caused by the COVID-19 pandemic.^1^ An estimated 10 million people developed TB globally in 2019, of whom approximately one-third were never detected or offered appropriate treatment.^1^ This persistent case-detection gap comprises around 2.9 million cases each year and results in continuing community transmission and undermining national and global TB control efforts.^2^

Studies from high-incidence settings have shown that patients with TB frequently experience significant delays before obtaining a diagnosis.^3^ The accessibility and trusted role of pharmacies in local communities^4^ mean that patients frequently attend private pharmacies soon after developing symptoms of TB.^5–7^ In India, Vietnam and Uganda, between 40% and 60% of patients who were ultimately diagnosed with TB visited a private pharmacy prior to medical assessment and TB diagnosis.^3 8 9^ Although private pharmacies should play an important role in case detection and referral, inappropriate management often contributes to delayed diagnosis and TB-specific treatment. Pharmacies may also contribute to the development of antimicrobial resistance (AMR) if they dispense inadequate broad-spectrum antibiotics to patients with TB.^10^ Hence, private pharmacies have been identified as important stakeholders in both TB and AMR control efforts.^6^

Private sector engagement is a top priority of the WHO, which encourages national TB control programmes to develop strong public–private partnerships as a part of their End TB Strategy.^11^ The private sector plays an important role in Vietnam^1^ and in other middle-income countries in Southeast Asia with a high incidence of TB and drug-resistant TB.^1^ In Vietnam, private pharmacies account for two-thirds of health service encounters.^12^ A previous study has shown that a minority of patients in northern Vietnam were referred by private pharmacies to a medical facility capable of establishing a TB diagnosis.^13^

We aimed to evaluate the referral practices of private pharmacies in Vietnam for standardised patients (SPs) presenting with symptoms of presumptive TB and drug-resistant TB as unannounced ‘mystery shoppers’.

## Methods

### Study design and setting

**Figure 1:**
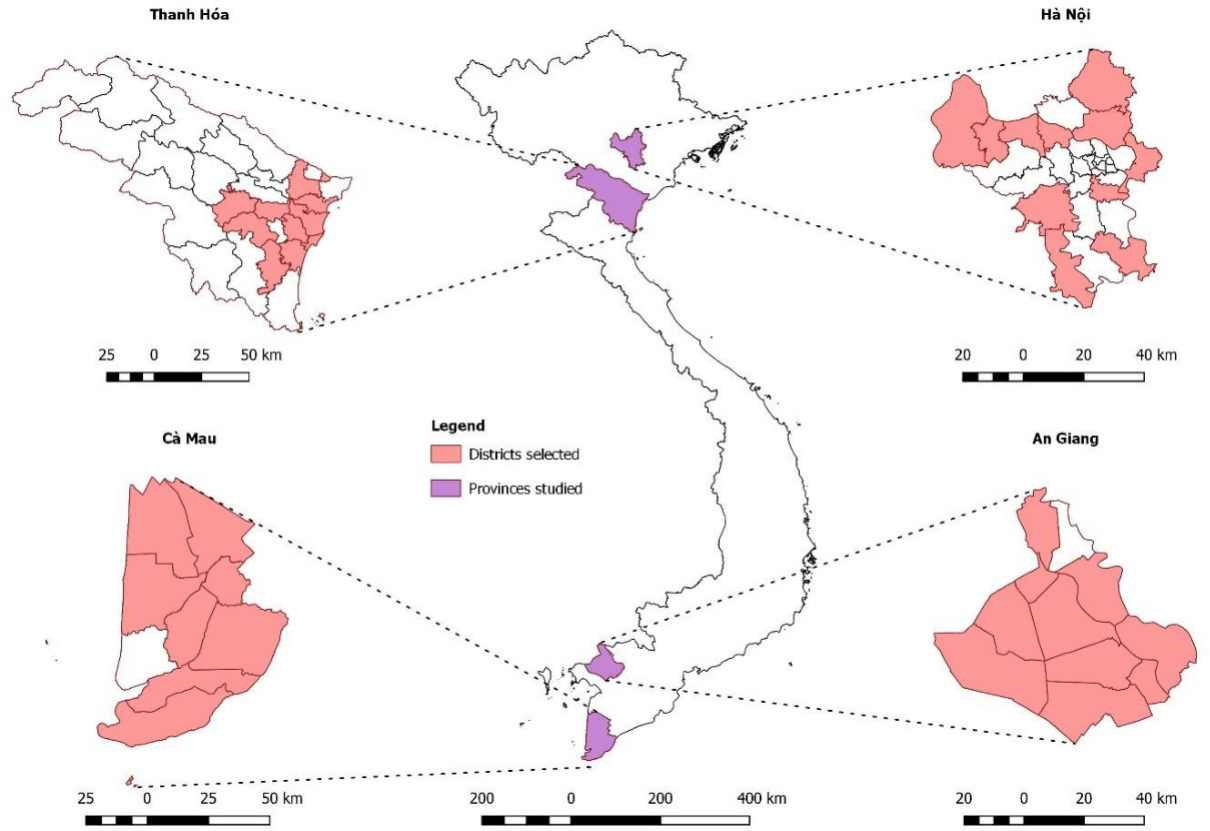
Provinces and districts selected for study participation.

A cross-sectional study was undertaken from April to November 2019 using an SP study method, with SPs visiting randomly selected private pharmacies in 40 districts of Vietnam. This included urban and rural areas in the northern (Hanoi and Thanh Hoa) and southern (Ca Mau and An Giang) provinces (figure 1). Vietnam is a middle-income country in Southeast Asia with a high incidence of TB (176/100 000) and multidrug-resistant TB (MDR-TB) (8.8/100 000) (defined as TB that is resistant to the two most effective first-line antibiotics, rifampicin and isoniazid).^1^ Tuberculosis diagnosis and treatment in Vietnam are largely provided by a public National Tuberculosis Program. However, the private pharmacy sector has expanded rapidly over the past two decades and is the first point of healthcare contact for the majority of patients.^12^

The SP study is regarded as a proven and widely used methodological tool for pharmacy practice research.^14 15^

#### Sampling

Districts located within 2 hours’ drive or less than 150 km from the provincial centre were included. Within each district, a geographical area comprising approximately one-quarter of the total population was selected by convenience sampling. The Global Positioning System location of private pharmacies in the selected geographical area was established by the research team physically walking the streets and identifying all pharmacies. Pharmacies were classified as either private pharmacies or drug counters. Vietnamese regulations stipulate that ‘private pharmacies’ in urban areas must be managed by a university graduate pharmacist, and ‘drug counters’ must be led by a pharmacist with college certification.^16^ Counters selling only traditional medicines, wholesalers and pharmacies located within government health facilities or private hospitals were excluded. Throughout this article, the term pharmacy refers to both drug counters and private pharmacies. Pharmacies were classified as registered (registration identification (ID) displayed on the signboard of the pharmacy) or unregistered (no ID displayed).

A total of 1643 eligible private pharmacies were identified during the mapping process. Details of the study site selection process are included in the online supplemental material.

10.1136/bmjgh-2021-006475.supp1 Supplementary data

### Development of clinical scenarios

Two clinical scenarios were developed in collaboration with clinicians, pharmacists, public health experts and researchers from Vietnam and Australia:

- *Presumptive TB:* the first scenario involved a male adult with the following symptoms: cough for 3 weeks, fever, loss of appetite and weight loss, as well as reporting a history of using cough medication without any improvement. The patient had not previously sought or received testing for TB.
- *Presumptive MDR-TB*: the second scenario comprised a patient with a cough of 4-week duration and a history of previous TB treatment, with early treatment interruption. The scenarios are summarised in table 1.

**Table 1:** Overview of SP scenarios

Scenario description	Presenting history	Expected outcome
1. Presumptive TB: 2–3 weeks of cough with intermittent fever, along with loss of appetite and weight loss; seeking medical advice for the first time.	‘I have a cough and fever for the last 2–3 weeks that is not getting better. I have lost my appetite and feel I am losing weight. I have taken cough medicine but there is no improvement. Can I get some medicines?’	Verbal or written referral to a doctor in a TB centre, hospital or a healthcare provider without dispensing any antibiotics.
2. Presumptive MDR- TB: 4 weeks of cough with history of incomplete TB treatment, seeking medical advice for the first time.	‘I have been suffering from a bad cough for about 4 weeks. I took some traditional medicines for cough relief, but they are not working. It seems to be similar to the cough I had 2 years ago when I got TB. But I remember that I was already treated for TB for 3 months and the tests were fine. Is it related to my previous condition? What do you suggest?’ The SPs would admit to a loss of appetite, to his clothes becoming a bit loose, fever and feeling fatigued if prompted by the pharmacy staff. If the staff asked about history of TB treatment, the SPs would say ‘I did it for about 3 months, later, when the doctor found my sputum test was fine, I thought it was cured and after 3 months of treatment, I stopped taking the medication’.	Verbal or written referral to a doctor in a TB centre, hospital or a healthcare provider without dispensing any antibiotics.

MDR-TB, multidrug-resistant tuberculosis; SP, standardised patient; TB, tuberculosis.

These two scenarios reflect typical clinical presentations with TB. Scenarios used in other settings^17 18^ were localised to the Vietnamese context, in consultation with clinical pharmacy lecturers, community pharmacists and other clinicians. The scenarios were piloted in 14 pharmacies in northern and southern regions, with further refinement prior to implementation.

For both scenarios, the research design included only middle-aged men as SPs because 75% of prevalent TB cases in Vietnam are male.^19^ Local actors were trained to present as an SP for one of the two scenarios over a 4-day period by a researcher with experience implementing SP studies in other settings. Training included initial briefing, interactive discussions, role plays and pilot visits to private pharmacies. Actors were evaluated against a checklist to ensure a convincing and standardised performance.

### SP visits

A sample of 636 pharmacies were allocated to each of the four provinces according to proportionate to the provincial sample of the eligible mapped pharmacies. A systematic sampling technique was used to select the pharmacies for each scenario. The fifth and sixth pharmacies were selected, starting from a random point of the list of eligible pharmacies in each province for presumptive TB and presumptive MDR-TB scenarios, respectively, until the provincial quota was filled. The same process was continued in all four provinces to randomly allocate pharmacies to both scenarios. The trained SPs were randomly assigned to pharmacies using a predetermined randomisation protocol.

Each selected pharmacy was visited during business hours by one SP and one observer. The observer was identified as a friend and listened to the interaction but did not participate in the clinical encounter. Immediately after leaving the pharmacy, the SP and observer jointly documented the outcome of the visit by completing a structured data collection sheet. They also collected an audio recording of their visit for narrative analysis. The recording mentioned the number of customers present in the shop, the number of staff present, the behaviour of the staff and the layout of the pharmacy.

#### Stepped antibiotic request

During each encounter, we assessed the pharmacy staff member’s willingness to supply antibiotics at three levels of increasing consumer demand.^14 20^ During the initial consultation, SPs asked for an unspecified medicine to alleviate their symptoms, without specifying an antibiotic (a ‘level 1’ request). If an antibiotic was not provided during the initial interaction, they asked for a stronger medicine: ‘can you give me any stronger medicine?’ (level 2 request). If the pharmacy staff did not provide an antibiotic, they then explicitly requested an antibiotic: ‘I want an antibiotic’ (level 3 request).

Medicines supplied by the pharmacy were identified by a team of three study pharmacists and were coded according to their therapeutic mode of action. Antibiotics were classified according to their generic name and grouped into three major categories: (1) fluoroquinolones; (2) non-fluoroquinolone broad-spectrum antibiotics (antibiotics that are effective against both Gram-positive and Gram-negative bacteria, including cephalosporins, broad-spectrum penicillins, sulfonamides, macrolides and tetracyclines); and (3) other antibiotics. We classified steroids and non-steroidal anti-inflammatory drugs as anti-inflammatory.

We assessed antibiotic supply in two ways: (1) ‘antibiotic offered’, which was reported in the data collection sheet by the SP and the observer (without medical knowledge) based on the interaction with pharmacy staff; and (2) ‘actual antibiotic supplied’, which was recorded after verification of the received antibiotics by the study pharmacists.

### Outcome measures

The primary outcome for both clinical scenarios was the proportion of ‘SPs’ who were referred for medical assessment. This referral includes to TB clinics, public hospitals and private clinics. Secondary outcomes included the proportion of SPs referred without antibiotic supply, the proportion pharmacy staff who asked for additional information about medical history and medicines supplied (online supplemental material describes predesigned list of additional information related questions).

### Data collection and analysis

#### Data collection

Data were documented on paper data collection forms immediately after a pharmacy visit and were later entered into an electronic Research Electronic Data Capture (REDCap) database designed without having any pharmacy identifiable information.^21^

#### Sample size

The sample size was based on an expected proportion of inappropriate antibiotic dispensing of 50%, based on studies from comparable settings.^17 22^ In order to ascertain the proportion of patients referred without supplying an antibiotic, with a precision of ±5% and an alpha of 0.05 (ie, 95% confidence limits), assuming half of the clusters were urban, we estimated that we needed a sample of 318 pharmacies for each scenario. This estimation was derived using sample size formula for proportions.^23^ Pharmacies were selected for each scenario from the eligible pharmacies using simple random sampling technique (online supplemental material).

#### Statistical analysis

Descriptive statistics evaluated pharmacy characteristics (classification of pharmacy, registration status, geographical area and gender of the pharmacy staff), duration of interaction, population density, pharmacy density, the medical history taken by pharmacy staff, medications supplied and referral practices. Logistic regression was used to evaluate the relationship between these independent variables and the primary outcome measures. The comparative analysis employed multivariable regression with backward elimination (SPSS V.24). Adjusted ORs (aORs) with 95% CIs were reported. The χ^2^ likelihood ratio test, pseudo R^2^ values and the Hosmer-Lemeshow χ^2^ test were used as model fit statistics to evaluate the multiple logistic regression models.

### Patient and public involvement

This SP study was designed to assess presumptive TB patient referrals and medication supply at community pharmacies. Therefore, study participants were pharmacy staff; patients or the public were not involved in the design, conduct, reporting or dissemination plans of our research.

## Results

Standardised patients visited 638 pharmacies, including 318 with presumptive TB and 320 presumptive MDR-TB. Characteristics of selected pharmacies are shown in table 2. A greater proportion of pharmacies were classified as drug counters (89.3%) than private pharmacies (10.7%). About 60% of the pharmacies displayed their registration status externally.

**Table 2:** Characteristics of participating pharmacies

Characteristics	SPs with presumptive TB	SPs with presumptive MDR-TB
	Frequency (%)	Frequency (%)
	N=318	N=320
Classification of pharmacy		
Pharmacy	30 (1.6)	38 (11.9)
Drug counter	288 (90.6)	282 (88.1)
Pharmacy registration status		
Not registered	129 (40.6)	128 (40.0)
Registered	189 (59.4)	192 (60.0)
Region		
North	200 (62.9)	201 (62.8)
South	118 (37.1)	119 (37.2)
Geographical area		
Rural	273 (85.8)	275 (85.9)
Urban	45 (14.2)	45 (14.1)
Gender of pharmacy staff attending the SP		
Male	73 (23.0)	65 (20.3)
Female	245 (77.0)	255 (79.7)
Duration of interaction (min)		
Median (IQR)	4 (3–5)	5 (4–5)
Missing data	1	
Commune population density (PPH)		
Median (IQR)	12 (8–16)	12 (8–17)
Number of pharmacies /10 000 population		
Median (IQR)	11 (7–17)	11 (7–17)

MDR-TB, multidrug-resistant tuberculosis; PPH, number of people per hectare; SP, standardised patient; TB, tuberculosis.

Among 318 SPs presenting with presumptive TB, 39 (12.3%, 95% CI 8.9% to 16.4%) encounters resulted in a referral to a healthcare provider (primary outcome). Among 320 SPs with presumptive MDR-TB, 116 (36.3%, 95% CI 31.0% to 41.8%) encounters resulted in a referral to a healthcare provider (primary outcome). Only 17 SPs (5.3%, 95% CI 3.1% to 8.4%) with presumptive TB and 68 (21.1%, 95% CI 16.9% to 26.1%) with presumptive MDR-TB were referred to healthcare providers without the provision of any antibiotics. Antibiotics were mostly offered without pressure (level 1 request) for the presumptive TB scenario by 92.3% of pharmacies, and for presumptive MDR-TB by 96.9% of pharmacies as compared with level 2 and level 3 requests (<8%).

Table 3 presents factors associated with referral for medical assessment. Pharmacies in the south were more likely to refer the SPs to healthcare providers than their northern counterparts for presumptive TB (aOR=8.8, 95% CI: 3.8 to 20.4) and presumptive MDR-TB (aOR=4.8, 95% CI 2.9 to 7.8), respectively. A significantly higher proportion of pharmacy staff inquired about the medical history of patients, during interactions with SPs who had presumptive MDR-TB (n=245, 76.6%), in comparison to those who had presumptive TB (n=195, 61.3%).

**Table 3:** Factors associated with referral of SPs with presumptive TB or MDR-TB for medical assessment

Predictors	Presumptive TB				Presumptive MDR-TB			
	N=318				N=320			
	Referred to healthcare provider (yes) Frequency (%)	Referred to healthcare provider (no) Frequency (%)	Unadjusted OR (95% CI) (referred compared with not referred)	aOR (95% CI) (referred compared with not referred) n=317 (model 1)	Referred to healthcare provider (yes) Frequency (%)	Referred to healthcare provider (no) Frequency (%)	OR (95% CI) (referred compared with not referred)	aOR (95% CI) (referred compared with not referred) n=319 (model 2)
Region								
North	9 (23.1)	191 (68.5)	1.0	1.0	46 (39.7)	155 (76.0)	1.0	1.0
South	30 (76.9)	88 (31.5)	7.2 (3.3 to 15.9)	8.78 (3.8 to 20.4)	70 (60.3)	49 (24.0)	4.8 (2.9 to 7.9)	4.7 (2.9 to 7.8)
Geographical location								
Rural	37 (94.9)	236 (84.6)	1.0	NI	95 (81.9)	180 (88.2)	1.0	NP
Urban	2 (5.1)	43 (15.4)	0.3 (0.1 to 1.3)		21 (18.1)	24 (11.8)	1.7 (0.9 to 3.1)	
Medical history taken by pharmacy staff								
No	10 (8.1)	113 (91.9)	1.0	1.0	20 (17.2)	55 (27.0)	1.0	1.0
Yes	29 (14.9)	166 (85.1)	2.0 (0.9 to 4.2)	3.9 (1.7 to 9.0)	96 (82.8)	149 (73.0)	1.8 (1.0 to 3.1)	1.8 (1.0 to 3.2)
Number of pharmacies/10 000 commune population								
Median (IQR)	10.7 (5.5–16.6)	10.7 (6.9–17.7)			10.6 (5.9–16.1)	10.8 (6.8–18.3)		NP
Low (<median)			1.0	NP			1.0	
High (≥median)			0.7 (0.9 to 1.7)				0.9 (0.6 to 1.5)	
Gender of attending staff								
Female	29 (74.4)	216 (77.4)	1.0	NP	86 (74.1)	169 (82.8)	1.0	NP
Male	10 (25.6)	63 (22.6)	1.2 (0.5 to 2.6)		30 (25.9)	35 (17.2)	1.7 (1.0 to 2.9)	
Registration status				NP				
Not registered	6 (15.4)	123 (44.1)	1.0		30 (25.9)	98 (48.0)	1.0	NP
Registered	33 (84.6)	156 (65.9)	4.3 (1.8 to 10.7)		86 (74.1)	106 (52.0)	2.6 (1.6 to 4.4)	
Type of facility				NI				
Pharmacy	1 (2.6)	29 (10.4)	1.0		16 (13.8)	22 (10.8)	1.0	NP
Drug counter	38 (97.4)	250 (89.6)	4.4 (0.6 to 33.3)		100 (86.2)	182 (89.2)	0.8 (0.4 to 1.5)	
Interaction time (min)				NP				NP
Median (IQR)	4 (4–5)	4.0 (3–5)						
Missing values	0	1			4.5 (4–5)	5.0 (4–5)		
Low (<median)			1.0				1.0	
High (≥median)			2.5 (1.1 to 5.9)				0.9 (0.6 to 1.5)	
Commune population density (PPH)								NP
Median (IQR)	8.4 (4.5–11.6)	12.2 (8.0–17.1)			10.6 (6.1–16.2)	12.6 (8.7–17.1)		
Low (<median)			1.0	1.0			1.0	
High (≥median)			0.3 (0.1 to 0.6)	0.4 (0.2 to 0.9)			0.6 (0.4 to 0.9)	

Model accuracy and fit: model 1: PAC=87.7%, omnibus χ^2^ test for the model <0.001 and Hosmer and Lemeshow χ^2^ test for the model=0.581.

Model 2: PAC=71.2%, omnibus χ^2^ test for the model <0.001 and Hosmer and Lemeshow χ^2^ test for the model=0.840. All of these values indicate that the models fit well.

aOR, adjusted OR; CI, 95% Confidence Interval; MDR-TB, multidrug-resistant tuberculosis; NI, not included in the adjusted model due to few/no observations found in one cell or few cells; NP, not predicted in the dimension of final model produced using backward deletion method; PAC, percentage accuracy in classification; PPH, number of people per hectare; SP, standardised patient.

Pharmacy staff who asked about SPs’ medical history were more likely to refer them to healthcare providers, for both the presumptive TB (aOR=3.9, 95% CI 1.7 to 9.0) and MDR-TB (aOR=1.7, 95% CI 0.9 to 3.3) scenarios. There was no association between the duration of the SPs’ encounter and the proportion of SPs referred for medical assessment.

Table 4 shows the categories of medicines supplied by pharmacy staff. A total of 1475 individual medicines were supplied during the presumptive TB encounters (median five drugs, IQR 4–6 drugs) and 1128 different medicines during the presumptive MDR-TB encounters (median four drugs, IQR 0–5 drugs). Over a quarter of medicines were unlabelled. Antibiotics were the most common category of medicines offered to SPs with presumptive TB (286/318; 89.9%, 95% CI 86.1% to 93.0%) and presumptive MDR-TB (225/320; 70.3%, 95% CI 65.0% to 75.3%).

**Table 4:** Pharmacy responses to SPs

Total SP scenarios	Presumptive TB	MDR-TB
	Frequency/proportion (%)	Frequency/proportion (%)
	N=318	N=320
Medical history taken*		
No	123 (38.7)	75 (23.4)
Yes	195 (61.3)	245 (76.6)
Referral to medical facility		
Total referrals (any of the following)	39 (12.3)	116 (36.3)
To TB centre	07 (2.2)	57 (17.8)
To private clinic	12 (3.8)	28 (8.8)
To district healthcare centre/hospital	31 (9.7)	46 (14.4)
Referral without giving antibiotics (subgroup of all aforementioned referrals)	17 (5.3)	68 (21.3)
Number of ‘all medications’ dispensed		
Median (IQR)	5 (4–6)	4 (0–5)
0 (no other medicine dispensed)	24 (7.5)	86 (26.9)
1	2 (0.6)	1 (0.3)
2	2 (0.6)	4 (1.3)
3	30 (9.4)	23 (7.2)
4	66 (20.8)	64 (20.0)
5	105 (33.0)	83 (25.9)
≥6	89 (27.9)	59 (18.4)
Categories of medicines dispensed		
Number different types of medicines received in total	1475 (100)	1127 (100)
Unlabelled medicines	436 (29.6)	311 (27.6)
Antibiotics (general)	244 (16.5)	217 (19.3)
Antibiotics (with anti-TB activity)	14 (0.9)	24 (2.1)
Cough medicine	230 (15.6)	207 (18.4)
Steroids/NSAIDS (anti-inflammatory)	113 (7.7)	107 (9.5)
Antihistamines	64 (4.3)	51 (4.5)
Pain/fever relief	46 (3.1)	16 (1.4)
Vitamin supplementary	70 (4.7)	32 (2.8)
Other identified medicine (bronchodilators, proton-pump inhibitors, etc)	106 (7.2)	81 (7.2)
Any combined formulation (combination of cough and cold formulations that included antitussives, antihistamines and/or analgesics)	152 (10.3)	81 (7.2)
Class of antibiotic dispensed†	N=258	N=241
Fluoroquinolones	45 (17.4)	39 (16.2)
Broad-spectrum antibiotics other than fluoroquinolones	202 (78.3)	190 (78.8)
Other antibiotic (includes lincomycins and unknown)	11 (4.3)	12 (5.0)
Number of pharmacies sold one antibiotic	238 (92.2)	196 (78.7)
Number of pharmacies sold two antibiotics	20 (7.8)	45 (18.7)
Prescription for an antibiotic requested by pharmacy staff		
No	312 (98.1)	212 (97.5)
Yes	6 (1.9)	8 (2.5)
Antibiotic offered‡		
No	32 (10.1)	95 (29.7)
Yes	286 (89.9)	225 (70.3)
Level of demand required to obtain antibiotic	N=286	N=225
Level 1 (Can I get some medicine to alleviate the symptoms?)		
Antibiotic given	264 (92.3)	218 (96.9)
Antibiotic not given	13 (7.7)	7 (3.1)
Level 2 (Can I get something stronger?)		
Antibiotic given	13 (4.5)	3 (1.3)
Antibiotic not given	9 (3.1)	4 (1.8)
Level 3 (I would like an antibiotic.)		
Antibiotic given	9 (3.1)	4 (1.8)
Antibiotic not given	0	0

*Possible history taken by staff in relation to reported symptoms.

†Actual antibiotics received after being verified by study pharmacists.

‡Reported by standardised patient prior to verification by study pharmacists but not necessarily bought by SP due to several reasons (eg, no change cash notes available, told that antibiotics was dispensed but could not find these and vice versa, etc).

MDR-TB, multidrug-resistant tuberculosis; NSAID, non-steroidal anti-inflammatory drug; SP, standardised patient; TB, tuberculosis.

### Antibiotics dispensed

Among the antibiotics verified by study pharmacists (n=258) during the presumptive TB encounters, 202 (78.3%) were broad-spectrum non-fluoroquinolone antibiotics (cephalosporins, broad-spectrum penicillins, macrolides, sulfonamides or tetracyclines) and 45 (17.4%) of antibiotics were fluoroquinolones. Medications used as part of standardised regimens to treat TB, such as levofloxacin or rifampicin, were supplied to 14 (5.4%) SPs with presumptive TB encounters and 33 (13.7%) SPs presenting with presumptive MDR-TB. No SPs were provided with a recommended treatment regimen for TB. More than one antibiotic was supplied in 16 (6.2%) and 39 (16.2%) encounters involving SPs with presumptive TB and presumptive MDR-TB, respectively.

Other common classes of medicines supplied to SPs were antitussives (16.8%), and combination cough and cold formulations (including two or more of antitussives, antihistamines and/or analgesics, 8.9%; table 4).

## Discussion

This SP study involving 638 pharmacies across Vietnam is the largest and most geographically representative study of its kind conducted in Vietnam to date. It demonstrated that only a small proportion of SPs with symptoms of presumptive TB and presumptive MDR-TB were referred for medical assessment. Pharmacists taking a medical history were more likely to make a referral than those who did not take a history. Broad-spectrum antibiotics, including fluoroquinolones, were commonly supplied to SPs.

The low proportion of referrals for medical assessment demonstrates the missed opportunities for early case-finding through pharmacies. Delays in accessing testing and treatment for TB undermine control efforts as patients may remain contagious for prolonged periods prior to diagnosis, increasing transmission of *Mycobacterium tuberculosis* in the community. Delayed diagnosis has been shown to increase TB-related morbidity and mortality.^24^

Our findings are consistent with studies using SPs in India and a self-reported study among pharmacy staff in Cambodia.^17 25^ In India, only 13.0% of the patients with presumptive TB symptoms and 62.0% of patients with confirmed TB were referred to a healthcare provider,^17^ whereas in Cambodia, 29.6% patients were referred for appropriate TB treatment.^25^ Our study found a lower proportion of referral across Vietnam than a study of 138 private pharmacies in an urban Hanoi province of Vietnam, in which 46.0% of patients were referred.^13^ In this prior study, 20 pharmacies located within 500 m distance from two TB hospitals were purposely selected and SPs specifically requested TB medications, potentially leading to more referrals. A cross-sectional study among patients with diagnosed TB in southern Vietnam found that those who visited a pharmacy prior to their diagnosis were five times more likely to have delayed access to care (which was defined as more than 4 weeks from first noticed symptom to first health provider contact), compared with those who consulted a national TB programme facility first.^26^

A higher proportion of referrals in the MDR-TB scenario were expected, as reported previous history of TB refrained from any differential diagnosis and supported by previous studies,^13 17^ only about one quarters of the pharmacies referred these SPs for screening. This poor referral suggests the pharmacy staff’s confusion or lack of knowledge in identifying presumptive MDR-TB cases. Educating pharmacy staff on TB-related symptoms and importance of presumptive TB case referrals might help to improve the early referrals for patients with presumptive TB.

There was significant regional variation in the referral patterns of pharmacies, with a greater referral rate in southern Vietnam. This shows a heterogeneity in private pharmacy practice between the regions, possibly due to various factors including, but not limited to, extent of pharmacy education and clinical training, business model and the culture that may contribute to this behaviour. This finding highlights the importance of undertaking studies in a wide range of settings in order to characterise differences in local practice and get a more comprehensive overview. Nevertheless, low case referrals were the norm in all settings, which indicates that further effort is required to enhance cooperation between the public and private sectors across the country.

Our study showed a high proportion of encounters which resulted in the supply of broad-spectrum antibiotics to SPs, including antibiotics used in TB treatment, particularly the fluoroquinolones (>16%) used in MDR-TB treatment. This is consistent with studies in other settings.^17 27^ These findings underpin concerns that unregulated dispensing of antibiotics is likely to amplify the risk of acquired drug resistance, especially against key second-line drugs.^28^ Inappropriate use of fluoroquinolones may also delay TB diagnosis due to a partial therapeutic effect that may improve patients’ symptoms and reduce the yield of microbiological tests for *M. tuberculosis*.^29^ Fluoroquinolones given to patients with undiagnosed MDR-TB compromise one of the most effective second-line TB drugs and will contribute to the emergence and spread of extensively drug-resistant TB.^30^ Antibiotics were dispensed predominantly at level 1 request. Similar findings were also observed in SP studies conducted at private pharmacies in Sri Lanka^14^ and Eritrea,^31^ where most antibiotics were spontaneously dispensed without any pressure exerted by SPs. Therefore, we hypothesise that the current inappropriate antibiotic dispensing practice may be reflect profit motive than external patient pressure. A previous study in Vietnam also found that the revenue of selling antibiotics without a prescription is greater than all sales in both rural and urban pharmacies.^32^ As we also found that over 95% of the sold antibiotics were broad-spectrum antibiotics, which are generally more expensive than narrow-spectrum antibiotics.

Our study has important public health implications by demonstrating a major opportunity to close persistent case-detection gaps if the private pharmacy sector can be better engaged. Our results demonstrate a need for interventions to improve the early recognition and appropriate referral of patients with presumptive TB (including MDR-TB) to public TB services. Training for pharmacy staff that improves their TB knowledge and emphasises the importance of history taking should improve the rate of referral. Doing this in a sustainable fashion may require inclusion in training curricula and better oversight/monitoring mechanisms. Support for effective public–private mix approaches, which have been advocated by the WHO and other international agencies, promise to make an important contribution to timely TB notification.^33^

The study also highlights the importance of improving antimicrobial stewardship (AMS) in general, especially in community settings where this is often neglected. AMS programmes promote the responsible use of antimicrobials through the delivery of evidence-based interventions but are often restricted to hospital settings.^34^ Although the programmes are highly regarded and considered essential to combat inappropriate antimicrobial use and protect human health in the long run,^35^ they are rarely implemented outside the hospital setting or with involvement of community pharmacies. This remains a major challenge requiring better solutions, especially in settings like Vietnam, where the vast majority of antibiotics are dispensed at the community level.

Our study has a number of strengths. First, the use of standardised patients provides an accurate assessment of the actual behaviour of pharmacy staff. This avoids social desirability bias and observer bias (the Hawthorne effect), which are difficult to avoid with self-reported methods. The selection of actors from local communities increased authenticity and reduced the risk of detection. Second, our study population was broadly representative of the situation in both northern and southern Vietnam. We randomly selected pharmacies for each scenario across 40 districts in the north and south of Vietnam, which adequately captured the diversity of practice across the study region.

Study limitations include the fact that the use of different actors may have introduced some variation in the scenarios. We reduced this by using a rigorous standardised training procedure with ongoing quality assessment through the presence of an observer and external monitoring. SP studies conducted in India found that the personal characteristics of SPs had little effect on the quality of care provided by staff.^36^ We included populations located within 2 hours of major urban centres in the north and south of the country. These were representative of urban and semirural populations in the most populous northern and southern regions of Vietnam. Remote rural populations were not examined in this study, for practical reasons. Our SPs had a restricted number of responses in order to improve standardisation across the study, although they were trained to communicate as naturally as possible. Responses were scripted using previous experience in conducting similar studies in other settings. While this study explored the practices of pharmacy staff, it did not assess the factors associated with pharmacists’ decisions to refer patients or provide antibiotics. This is an important area for future research, to enable interventions that will improve referral practices in the private pharmacy sector.

In conclusion, this study revealed that a low proportion of private pharmacies in Vietnam appropriately referred patients with presumptive TB for medical assessment. This is important to address, given the considerable TB case-detection gap observed in Vietnam,^19^ and highlights the importance of strengthening partnerships between the private and public sectors in order to achieve the WHO End TB targets. It also emphasises the need to involve community pharmacies in AMS initiatives in order to reduce the inappropriate use of broad-spectrum antibiotics in Vietnam.

## Data Availability

All data relevant to the study are included in the article or uploaded as supplementary information. All deidentified data are included in the article and tables.
