# Supplementary material for: Standardised patient study to assess tuberculosis case detection within the private pharmacy sector in Vietnam
Source: BMJ Glob Health. 2021 Oct 5;6(10):e006475. doi: 10.1136/bmjgh-2021-006475 (PMC8496389; doi:10.1136/bmjgh-2021-006475)
Supplement: Supplementary data [file bmjgh-2021-006475supp001.pdf]

Quality assurance

Quality assurance measures to ensure compliance with the study protocol included recruitment of SPs from the local population (to ensure a suitable local accent) and monthly observation of SPs by a senior researcher to ensure compliance with standard interaction procedures. Each pharmacy was selected and visited only once with a randomly selected scenario. Quantitative findings were verified against qualitative recordings.

Study sites

Vietnam is administratively divided into provinces, districts, and communes (wards for City level). We mapped the private pharmacies in four provinces, two from the north (Ha Noi and Thanh Hoa) and two from the south of Vietnam (An Giang and Ca Mau). Within each study province, we performed stratified sampling to select survey clusters. First, we selected districts, where they were eligible if (i) the district urban center is located no more than 2 hours by road or is less than 150km from the provincial urban center, as defined by Google Maps (Google LLC, USA). Next, we selected a sub-district cluster of adjacent communes comprising approximately one quarter of the total population of the district. Communes are ranked by population. The four most populous communes in each district were selected as seed communes. Sub-district clusters were selected by first identifying the commune with the largest population (seed commune), then selecting contiguous communes adjacent to the initial seed commune in a clockwise direction (towards the boundary of the district) until 25% of the district population was contained within the sub-district cluster. The second sub-district cluster included the successive communes adjacent to the second seed commune again in a clockwise direction. This process was repeated for the third and fourth sub-district clusters. If some seed communes were adjacent to each other, adjacent communes were selected such that the respective total population in each sub-district cluster did not exceed one quarter of the total district population. This procedure generated 4 sub-district units, each comprising approximately 25% of the total population of each district.

Each of these sub-districts became a randomization unit. We then randomly selected one sub-district cluster from each district using the minimization method. Randomization was performed by a person who is not otherwise engaged in the process and is blinded to the identity of the districts. We mapped all drug outlets in communes from within the selected mapping clusters from each study province.

Eventually we mapped 1972 private pharmacies and drug counters and 1643 were eligible for the standardised patient survey (SPS). The private pharmacies and drug counters are the places where medicines are sold and located outside the hospitals with a sign stating that they supply medicines. Total 638 pharmacies were randomly selected for SPS for the eligible mapped pharmacies. A proportionate to provincial sample was estimated based on number eligible mapped pharmacies and estimated sample of pharmacies required for the study, which was determined based on the total number of facilities in each province and total sample of pharmacies required. Simple random sampling technique was used to obtain a potentially representative and random sample of pharmacies from each province.

Zawahir S, *et al. BMJ Global Health* 2022; 6:e006475. doi: 10.1136/bmjgh-2021-006475

SP Recruitment, Training and Monitoring

For both scenarios, recruited men in their middle ages from north and south localities as standardised patients, because 75% of prevalent TB cases in Vietnam are male [16] and to acknowledge the different of Vietnamese language in these regions.

The locally recruited actors were trained to present as standardised patient one of the two scenarios over a four-day period, by a researcher with experience implementing standardised patient study in other settings.

The training was conducted on how to act in all scenarios, complete data collection forms, exit qualitative recording of the interactions in the pharmacy, labelling of medicines and handing over the purchased medicines and questionnaires to team leaders. Pursuant to the prepared information, the standardised patients were instructed to reply briefly to any questions from the pharmacy staff during the interaction, and to provide no additional information unless asked by them. The training also included indoor role plays and pharmacy pilot visits. The pilot visits were conducted outside the sample study pharmacies. The pilot visits ensured feasibility and accuracy of interactions based on developed scenarios and the script for the interactions. Standardised patients were evaluated against a checklist to ensure a convincing and standardised performance.

Either a research coordinator or the trainer performed monthly independent quality assessment visits to pharmacies to ensure each standardised patient adherence to the scenario and interaction guide provided to them. These visits were made concurrently as a separate customer to the pharmacy during the observed interaction. Standardised patients were provided with feedback about their performance following each quality assessment visit.

Zawahir S, *et al. BMJ Global Health* 2022; 6:e006475. doi: 10.1136/bmjgh-2021-006475

Pre-listed possible questions that could be asked by pharmacy staff

**Scenario type**	**Presumptive TB**
**Other possible detail could be asked by pharmacy staff**	**Responses (If asked by pharmacy staff only)**
1. What is the duration of cough?	1. 2-3 weeks, mainly during the morning and at night
2. Does anything bring up when you cough?	2. Yes, yellowish sputum
3. Does blood come out when you cough?	3. No
4. Do you have any chest pain?	4. No
5. Do you feel lost of appetite?	5. I think yes
6. Do you feel any unintended weigh loss?	6. Yes, my cloths becoming a bit loose
7. Do you have fever?	7. Yes, can feel slight fever for 2-3 weeks
8. What type of fever do you have?	8. Mild, on and off specially in the evenings
9. Do you feel fatigue?	9. Yes, I do
10. Does anyone at home having similar conditions?	10. No
11. Do you feel chills at night?	11. I am not sure about that
12. Do you have night sweat?	12. I am not sure about that
13. Did you visit a doctor?	13. No
14. Do you use any medicine for the current condition?	14. Tried with cough syrup and paracetamol.
15. Have you been treated for TB in the past?	15. No
16. Anyone in your family has TB?	16. No
17. Do you smoke?	17. Yes, I smoke cigarettes
18. How many cigarettes per day?	18. About one pack
19. How long you have been smoking?	19. Last 8 – 10 years
20. Do you drink alcohol?	20. Yes, I drink once or twice a week
21. Do you have diabetes?	21. I do not know
22. Do you have high blood pressure?	22. I do not know
23. Do you have HIV-AIDS?	23. I do not think I did
24. Have you ever been tested for these diseases?	24. I do not think I did
25. All any other possible questions.	25. Should be answered directly (most probably negative response) without any detail.

Pre-listed possible questions that could be asked by pharmacy staff

**Scenario type**	**Presumptive MDR-TB**
**Other possible detail could be asked by pharmacy staff**	**Responses (If asked by pharmacy staff only)**
1. What is the duration of cough?	1. 3-4 weeks, mainly during the morning and at night
2. Does anything bring up when you cough?	2. Yes, yellowish sputum
3. Does blood come out when you cough?	3. No
4. Do you have any chest pain?	4. Yes
5. Do you feel lost of appetite?	5. I think yes
6. Do you feel any unintended weigh loss?	6. Yes, my cloths becoming a bit loose
7. Do you have fever?	7. Yes, can feel slight fever for 2-3 weeks
8. What type of fever do you have?	8. Mild, on and off specially in the evenings
9. Do you feel fatigue?	9. Yes, I do
10. Do you feel chills at night?	10. I am not sure about that
11. Do you have night sweat?	11. I am not sure about that
12. Did you visit a doctor?	12. No
13. Do you use any medicine for the current condition?	13. Tried some traditional medicines for cough.
14. Have you been treated for TB in the past?	14. Yes
15. When was it?	15. About 2 years ago
16. Have you visited TB clinic last time when you had TB?	16. Yes
17. Have you completed the medication given at the clinic last time when you had TB?	17. Yes I did for about 3 months, later, when doctor found my sputum test was fine, I thought it was cured and after 3 months of treatment, I stop taking the medication.
18. Have you attended TB clinic this time?	18. No
19. Anyone in your family has TB?	19. No
20. Do you smoke?	20. Yes, I smoke cigarettes
21. How many cigarettes per day?	21. About one pack
22. How long you have been smoking?	22. Last 8 – 10 years
23. Do you drink alcohol?	23. Yes, I drink once or twice a week
24. Do you have diabetes?	24. I do not know
25. Do you have high blood pressure?	25. I do not know
26. Do you have HIV-AIDS?	26. I think no

Zawahir S, *et al. BMJ Global Health* 2022; 6:e006475. doi: 10.1136/bmjgh-2021-006475

27. Have you ever been tested for these diseases?<br><br>28. All any other possible questions.	27. I think no<br><br>28. Should be answered directly (most probably negative response) without any detail.
